# Root Canal Treatment of a Two-Rooted C-Shaped Maxillary First Molar: A Case Report

**Published:** 2014-10-07

**Authors:** Sara Paksefat, Saeed Rahimi

**Affiliations:** a *Department of Endodontics, Dental and Periodontal Research Center, Tabriz Dental School, Tabriz University of Medical Sciences, Tabriz, Iran*

**Keywords:** C-shaped, Maxillary First Molar, Root Canal Anatomy, Root Canal Treatment

## Abstract

The most difficult maxillary teeth for endodontic treatment are the maxillary first molars (MFM) due to their complex root canal anatomy. The presence of two roots and C-shaped canals in MFMs has been reported in rare cases. The present case reports root canal treatment of MFM with two roots, where the palatal and buccal roots were joined together in a C-shaped configuration.

## Introduction

Thorough clinical knowledge about the exact anatomy of each tooth and normal variations in the root canal system is a prerequisite for successful root canal treatment (RCT)  [1] . Maxillary first molars (MFM) are among the most difficult teeth for endodontic treatment due to their complex root canal system [2]. Therefore, they have been the subject of many studies carried out on their morphology, root canal system and the anatomic variations. The most commonly reported anatomic configuration for the MFMs is the presence of 3 roots (two buccal and one palatal) and 4 root canals. Usually, there are two canals in the mesiobuccal (MB) root and one canal is located in each of the distobuccal (DB) and palatal (P) roots [3, 4]. Several laboratory and clinical studies have reported specific variations in the anatomy of these teeth, regarding the number of roots and root canals and the pulp chamber configuration [2, 5-8]. Variations such as presence of one, two, four and five roots have been reported [9-11]. Regarding the pulp chamber configuration and canal orifices, one of the rarest anatomic variations in these teeth is the C-shaped configuration [12].

The present case report presents the endodontic treatment of a permanent MFM with two roots and a C-shaped anatomy.

## Case Report

A 27-year-old female referred to the Department of Endodontics, Tabriz Faculty of Dentistry, for endodontic treatment of the left MFM .The patient complained of a carious lesion on the tooth for two years and pain on mastication for the last 2 months. Her medical history was non-contributory.

Clinical examination revealed an amalgam restoration with recurrent carries and an orthodontic bracket on the left MFM. The patient had undergone orthodontic treatment of maxillary jaw for the past 2 years, which was deactivated 4 months earlier.

The tooth responded normally to percussion and palpation tests. The gingival attachment apparatus was normal, as well. The tooth exhibited a heightened response to thermal tests compared to control teeth. A diagnosis of irreversible pulpitis was reached based on the results of thermal tests and RCT was suggested to the patient. An informed consent was also obtained from the patient.

**Figure 1A:**
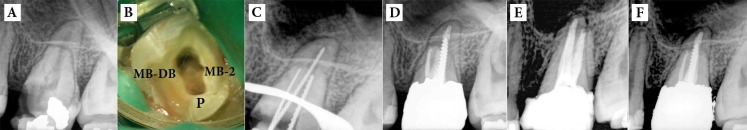
*A*) Preoperative radiograph of the left maxillary first molar; *B*) The access cavity showing the palatal (P) and second mesiobuccal (MB-2) canal orifices and fused mesiobuccal (MB) and distobuccal (DB) canal orifices confirming a C-shaped anatomy; *C*)Working length determination;

Close examination of the periapical radiograph revealed the presence of two roots (Figure 1A). Supplemental radiographs were taken at different horizontal angulations to confirm this anatomy. The tooth was anesthetized with buccal infiltration of 2% lidocaine containing 1:80000 epinephrine (DarouPakhsh, Tehran, Iran) and was isolated with a rubber dam.

*D*) Final radiography after root canal obturation; *E*) The 3-month follow-up radiography; *F*) The 6-month follow-up radiography

**Figure 2:**
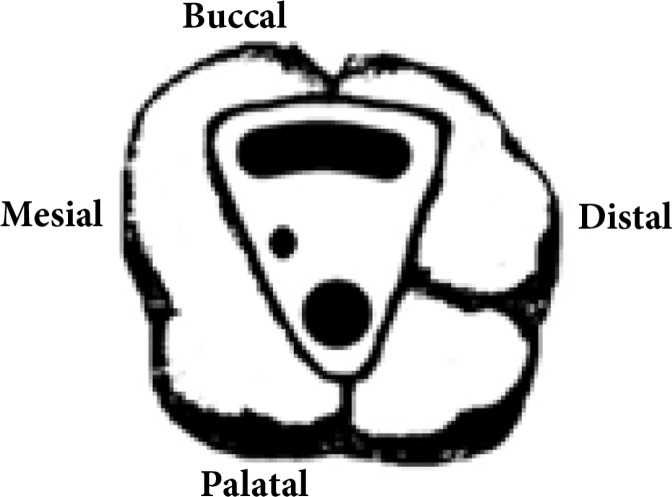
Schematic image of the treated case showing C-shaped orifices and a separate second mesiobuccal canal (Type B)

After caries removal, an access cavity was prepared and three canal orifices were identified: one large ovoid orifice which occupied the entire buccal portion of the pulp chamber, one orifice at the palatal area and another orifice where we were expecting to locate the orifice of the second mesiobuccal root canal orifice (Figure 1B). The pulp chamber and the orifices were more accurately evaluated under a dental operating microscope (Seiler Revelation, Seiler Instruments, St Louis, MO, USA).

The buccal root canal had a C-shaped configuration and occupied the entire buccal segment of the pulp chamber. The working length (WL) was estimated using Root ZX apex locator (J. Morita USA, Inc., Irvine, CA, USA). A radiograph was taken to confirm the WL (Figure 1C). Mechanical preparation of the root canals was done by hand K-files (Dentsply Maillefer, Ballaigues, Switzerland) with step-back technique. The buccal root canal was very wide without any isthmus. This root canal was circumferentially instrumented by Hedström files (Dentsply Maillefer, Ballaigues, Switzerland); 1.3% NaOCl and saline were used for repeated irrigation. The root canals were filled with creamy calcium hydroxide (CH) paste (Golchay, Tehran, Iran) for one week.

In the second session, the patient’s symptoms had subsided completely. After washing the CH from the root canals, they were once again irrigated with 1.3% NaOCl and normal saline. The root canals were dried with paper points (Ariadent, Tehran, Iran) and sizes 80, 45 and 30 gutta-percha master cones (Ariadent, Tehran, Iran) were placed in B, P and MB-2 root canals, respectively. Then confirmation radiography was taken. Lateral compaction technique was used to obturate the root canals with gutta-percha and AH-26 sealer (Dentsply, De Trey, Konstanz, Germany). The final radiograph showed complete obturation of the wide buccal canal (Figure 1D) and the tooth was referred for permanent restoration (Figure 1E and F).

## Discussion

This report presented the endodontic treatment of a MFM with one of the rarest anatomies consisting of 2 roots with a C-shaped configuration. The endodontic literature has described the permanent MFM as a tooth mostly with 3 roots and 4 canals [3]; variations with 1 to 6 roots and 1 to 8 root canals have also been reported [13]. The presence of 2 roots is very rare. A total of 7 studies evaluating 1629 MFMs regarding the odds ratio of fused roots in these teeth have reported that fusion occur in ~5.2% of these teeth, the majority of which include DB and P roots, with a lower incidence of MB and DB roots [14]. Fusion of two buccal roots in double-rooted MFMs occurs in almost 4% of cases [15]. Another rare variation is the C-shaped canal configuration [12]. C-shaped canals were first reported by Cooke and Cox in a mandibular second molar in 1979 [16]. In a review by Clegorn *et al.* [14] on the root morphology of MFMs, the prevalence of C-shaped canals was reported to be 0.12%.

Sabala *et al.* [17] carried out a study on the bilateral prevalence of anatomic variations of teeth and reported that the rarer is the variation the more are the chances of its bilateral occurrence. However, in the case presented here the contralateral MFM exhibited normal root canal anatomy. In 1991, Melton *et al.* [18] introduced a classification for C-shaped root canal morphology based on the cross-sectional configuration, according to which the buccal root of the present case can be placed in category II (semicolon-shaped canal) where dentin separates one distinct canal in the same section.

The results of another review study by Martins *et al.* [13] showed that to date only 10 cases of C-shaped root canals have been reported in MFMs, in 6 teeth the C-shaped morphology was attributed to the fusion of DB and P roots (Type A) and in 3 it was due to the fusion of MB and DB roots, (Type B); two other cases consisted of fusion of two P roots, which is referred to as Type C. In the present case, the MFM had two roots and the root canals were C-shaped (Type B) based on the aforementioned classification *i.e.* fusion of the DB and MB roots. This case seems to be the first report of fused MB and DB roots (C-shaped) and a separate MB-2 root canal (Figure 2). 

## Conclusion

In maxillary first molars, fusion of mesiobuccal and distobuccal roots and a separate second mesiobuccal canal with a C-shaped configuration is very rare. Awareness of clinicians about the anatomy and variations of these teeth and meticulous attention during pulp chamber negotiation to locate all the canal orifices are absolutely necessary for successful endodontic treatment.
